# The value of intratumoral and peritumoral radiomics features based on multiparametric MRI for predicting molecular staging of breast cancer

**DOI:** 10.3389/fonc.2025.1379048

**Published:** 2025-03-11

**Authors:** Yuxuan Han, Manxia Huang, Lizhi Xie, Yuhai Cao, Yang Dong

**Affiliations:** ^1^ Department of Radiology, The Second Affiliated Hospital of Dalian Medical University, Dalian, China; ^2^ GE Healthcare, MR Research China, Beijing, China

**Keywords:** breast cancer, molecular typing, radiomics, magnetic resonance imaging, peritumoral

## Abstract

**Purpose:**

A model for preoperative prediction of molecular subtypes of breast cancer using tumor and peritumor radiomics features from multiple magnetic resonance imaging (mMRI) sequences, combined with semantic features.

**Materials and methods:**

A total of 254 female patients with pathogically confirmed breast cancer were enrolled in this study. Preoperative mMRI, including T2-weighted imaging (T2WI), diffusion-weighted imaging (DWI), and dynamic contrast-enhanced MRI (DCE) sequences, covered the entire breast. To analyze the MRI semantic features of different molecular subtypes of breast cancer and identify independent predictive risk factors. Thirty-three binary classification models were established based on the radiomic features of different sequences and peritumoral ranges. The best radiomics model was selected by comparing the performance of the above radiomics models. At the same time, the best sequence and peritumoral extent were extracted from the target features, the radiomics score was calculated, and independent risk factors were predicted. Finally, a nomogram was established for preoperative prediction of Triple-Negative Breast Cancer (TNBC), Hormone Receptor (HR) positive and HER2 negative (HR+/HER2−), and HER2+ molecular staging types of breast cancer.

**Results:**

Tumor length, edge enhancement, and peritumoral edema were independent risk factors for predicting the different molecular types of breast cancer. The best MRI sequence was DCE and the best peritumoral margin was 6 mm. The AUC of the nomogram based on the optimal sequence(DCE) and optimal peritumoral range (6 mm) combined with independent risk factors were 0.910, 0.909, and 0.845, respectively.

**Conclusion:**

The nomogram based on independent predictors combined with intratumoral and peritumoral radiomics scores can be used as an auxiliary diagnostic tool for molecular subtype prediction in breast cancer.

## Highlights

- Preoperative prediction of molecular subtypes of breast cancer is very important.
- Intratumoral and peritumoral radiomics features of breast cancer contain useful information.
- Nomogram may provide a tool for the prediction of molecular subtypes of breast cancer.

## Introduction

Breast cancer has become the most common cancer in women, is responsible for the largest number of cancer-related deaths, and is gradually increasing (1, 2). Molecular subtypes are used to determine the important basis of treatment. Hormone receptor-positive type (Estrogen Receptor (ER)+ or Progesterone Receptor (PR)+) can be treated with endocrine therapy, Human Epidermal growth factor Receptor2 (HER2)+ can be treated with targeted therapy with anti-HER2 monoclonal antibody, and all receptor deficiency types, namely triple negative type, are mainly treated with chemotherapy (3, 4). Preoperative non-invasive prediction of breast cancer molecules is an important indicator of the biological behavior and prognosis of breast cancer and provides valuable information for the formulation of neoadjuvant chemotherapy regimens and prognosis of breast cancer.

Currently, immunohistochemistry using surgical specimens is the main method for molecular subtyping of breast cancer, and is determined by the expression of ER, PR, and HER2. However, preoperative biopsy is invasive and time consuming. Due to the tumor heterogeneity, a sampling bias exists. Radiomics may provide a non-invasive method for the preoperative prediction of molecular subtypes of breast cancer and has become a hot topic in medical imaging research.

Radiomics research on breast cancer has made some progress, but the optimal sequence used to establish radiomics models for breast cancer molecular typing has not been fully compared (5, 6). Magnetic Resonance Imaging (MRI) signals in the peritumoral region of breast cancer can provide complementary imaging information to the intratumoral region, which can be used to evaluate microenvironmental characteristics such as peritumoral angiogenesis, lymphangiogenesis activity, lymphatic and vascular invasion, tumor tissue stromal reaction, and lymphocyte infiltration immune response (7, 8). However, the optimal peritumoral region for the evaluation of molecular subtypes of breast cancer has not been clarified (9). There is still room for further research on radiomics of the molecular subtypes of breast cancer.

The aim of this study was to explore the optimal MRI sequence and peritumoral range for the establishment of a predictive model for molecular classification of breast cancer, combined with the risk predictors in Breast Imaging Reporting And Data System (BI-RADS) evaluation of breast cancer, and to establish a radiomics nomogram, which can provide a reference for radiomics research on molecular classification of breast cancer.

## Materials and methods

### Patients and MRI acquisition

Approved by the ethics committee of our institution, this study retrospectively collected 363 female breast cancer patients who were admitted to our hospital between January 2019 and December 2021 and confirmed by postoperative pathology. Finally, we included 254 women who met the criteria for this study and were randomly divided into two datasets (178 in the training set and 76 in the validation set) at a ratio of 7:3 in the binary classification analysis. The inclusion and exclusion criteria for these cases are listed in **Supplementary Table 1**. All patients were scanned with three MRI scanners, including one 1.5 T MRI scanner (General Electric Signa HDxt) and two 3.0 T MRI scanners (General Electric Discovery MR 750 W and Siemens Verio). All patients were in the prone position and scanned using a matching bilateral breast-dedicated coil. All patients underwent T1-Weighted Imaging (T1WI), Fat Sat T2-Weighted Imaging (FS-T2WI), Diffusion-Weighted Imaging (DWI), and Dynamic Contrast-Enhanced (DCE) MRI examinations. Detailed parameters are listed in **Supplementary Table 2**.

### Analysis of MRI semantic features

MRI images were analyzed according to BI-RADS 2013 of the American College of Radiology by two radiologists with 3 years and 15 years of experience in breast MRI diagnosis, respectively. In cases of disagreement, a third radiologist (with 15 years of experience in breast MRI) made the final decision. The content and methods of the analysis are shown in **Supplementary Table 2**.

### Radiomics analysis

#### Segmentation of breast cancer lesions

**Figure 1:**
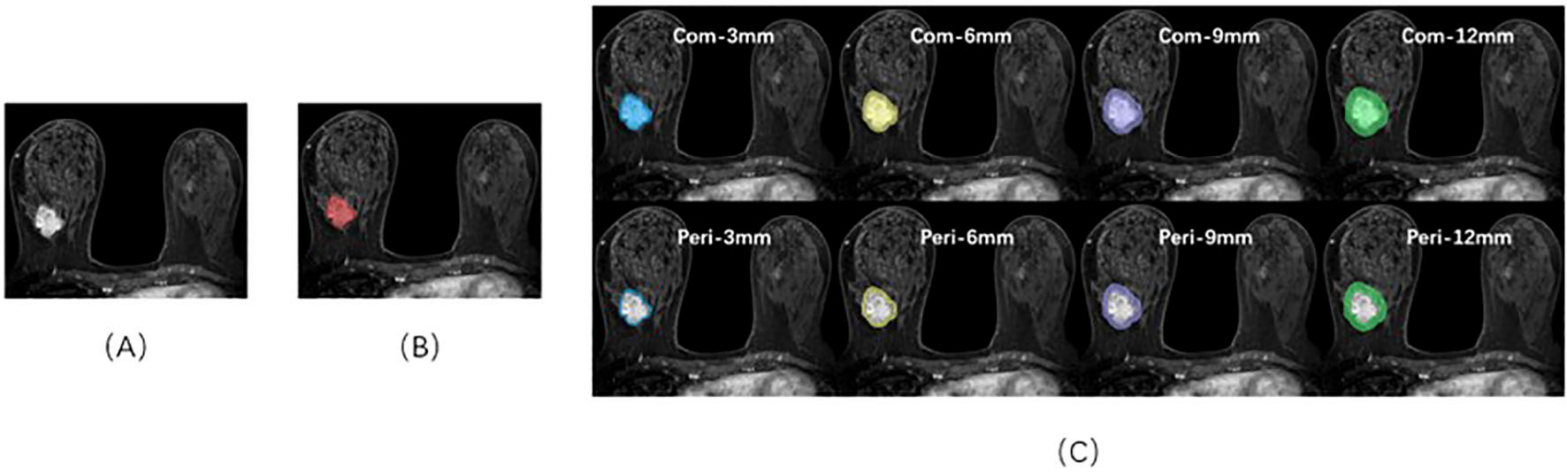
Sketch of the breast cancer ROI on DCE-MRI images using 3D-Slicer software. **(A)** A 46-year-old female patient with triple-negative breast cancer. **(B)** The red area represents the ROI of the tumor body. **(C)** ROIs of the tumor bodies and peritumoral and peritumoral regions in different ranges.

The FS-T2WI, DWI, and DCE images were imported into 3D Slicer (version 4.10.2) software and completed by two radiologists with 3 years and 15 years of imaging diagnosis experience, respectively. Three different Volume Of Interest (VOI) models were constructed by the following methods (**Figure 1**): Tumor model; The combined model of tumor and peritumoral: the range was selected to expand 3 mm, 6 mm, 9 mm, and 12 mm, which was represented by “com-;” Peritumoral model: denoted by “peri-.”

#### Radiomics feature extraction

The “radiomics” module in the open-source software 3D Slicer was used to preprocess and segment the images. Four categories were extracted from intratumoral and peritumoral VOI, including shape features, first-order features, texture features, and higher-order statistical features. A total of 1,130 quantitative radiomics features (ICC >0.75) and detailed features are shown in **Supplementary Table 4**, which were in accordance with the Image Biomarkers Standardization Organization (IBSI) standard (10). Before feature extraction, the MR Images of each sequence of all patients were resampled and voxels with different original sizes in medical images were normalized to the same size (11).

#### Dimensionality reduction of radiomics features

**Figure 2:**
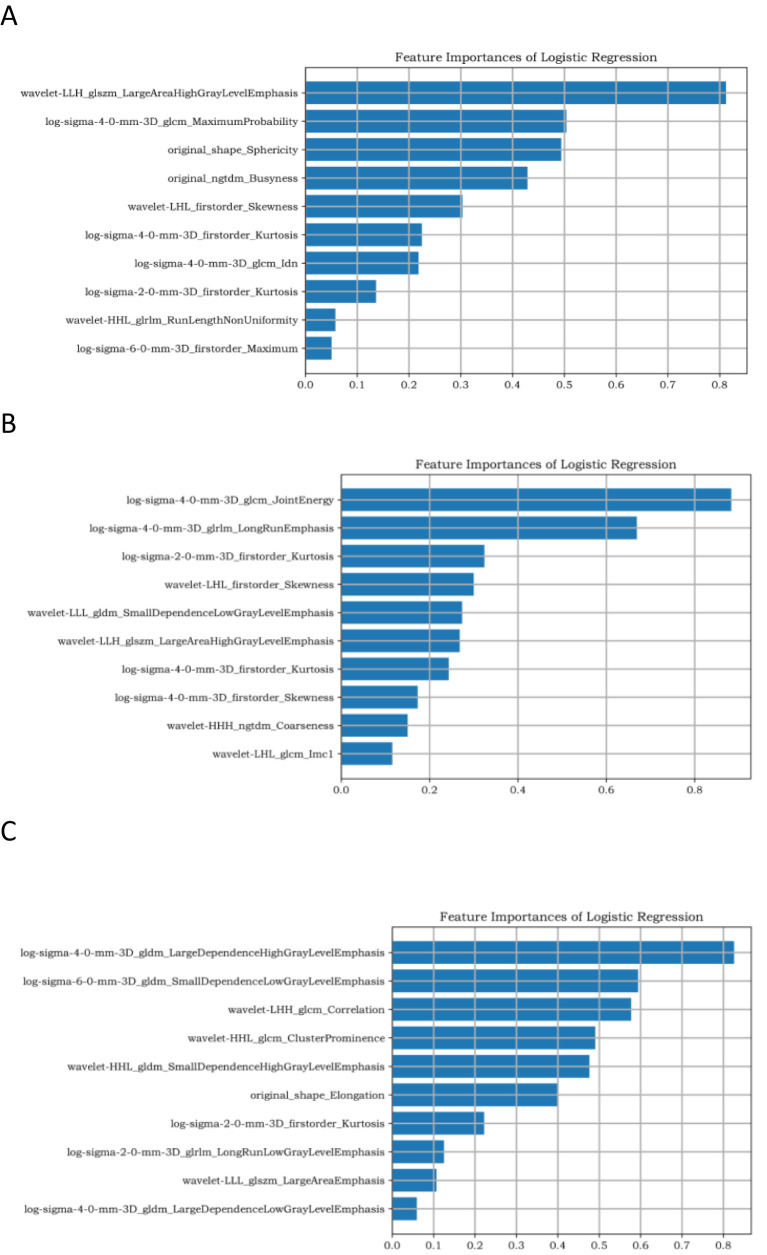
Ten target features based on DCE sequences. **(A–C)** represent the dimensionality reduction results of TNBC, HR+/HER2−, and HER2+ breast cancer, respectively.

Upload 1,130 features of the above-mentioned documents to the Yizhun–Darwin intelligence platform (http://premium.darwin.yizhun-ai.com/). To prevent the effect of size differences between features on feature selection, minimum and maximum normalizations were used to normalize the feature size, and all pixel values were normalized to 0 and 1. To avoid dimension disasters, we used two methods to gradually select the optimal features. First, the percentile selection method was used to select the top 10% of the most important features for classification. The variable with the highest correlation was then selected using the minimum redundancy maximum association, and redundant features with strong correlations between features were removed. After dimension reduction, 10 key features were retained for subsequent model training under the DCE, T2WI, and DWI sequences, as shown in **Figure 2**.

#### Construction of radiomics model

**Figure 3:**
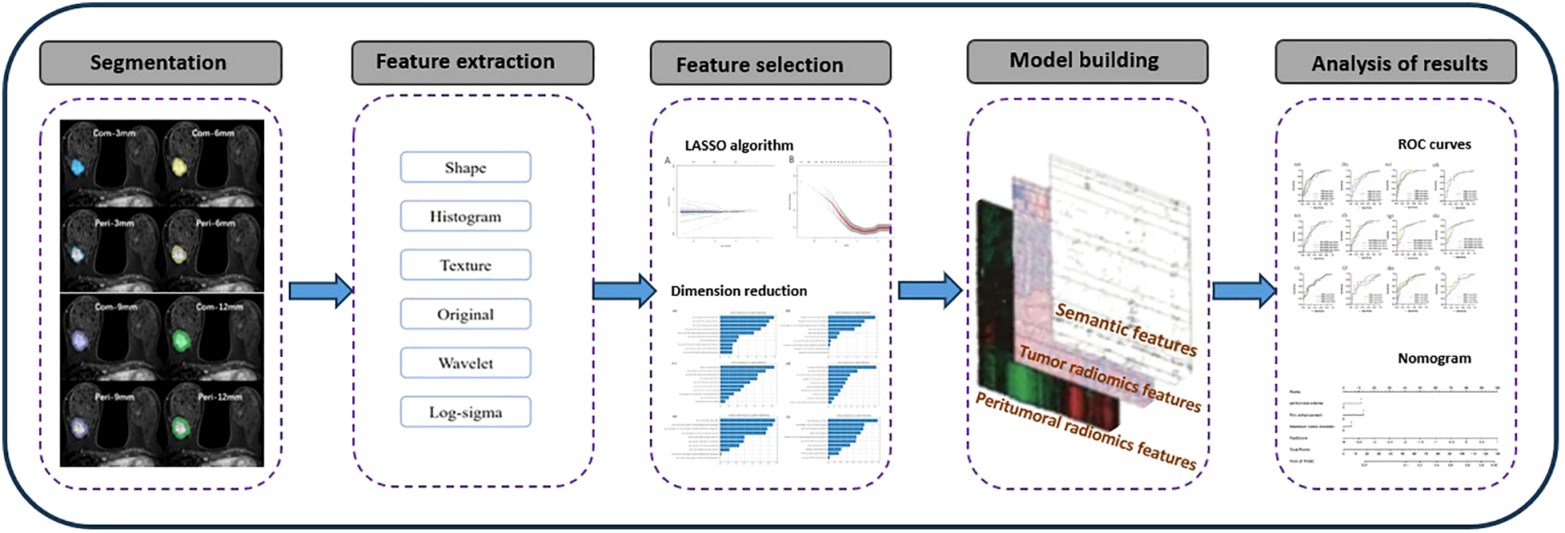
Flowchart of radiomics methods.

A logistic regression classifier was used to establish the radiomics model. First, an Optimization Function was constructed. The function of the Optimization Function is to adjust the corresponding parameters such that the Loss Function becomes increasingly smaller. The calculation method is usually the derivative of the Loss Function with respect to parameter. Through multiple-cycle training, the Loss Function value tends to be minimized, and the prediction effect tends to be the best. A receiver operating characteristic (ROC) curve was used to evaluate the prediction efficiency of the model. The area under the curve (AUC), 95% confidence interval (CI), accuracy, sensitivity, and specificity were also calculated. The flowchart is shown in **Figure 3**.

### Nomogram

Correlation analysis of the clinical information, MRI semantic features, and molecular subtypes of breast cancer was performed. Variables with statistically significant differences were included in univariate and multivariate logistic regression analyses to screen for independent predictors to distinguish the three groups of breast cancer molecular subtypes. The radiomics score (Rad-score) was calculated using Least Absolute Shrinkage and Selection Operator (LASSO) regression to construct the nomogram.

### Histopathological analyses

The expression of ER, PR, and HER2 in each patient was recorded using immunohistochemistry (IHC) and Fluorescence *In Situ* Hybridization (FISH). Ki-67 expression was defined as 14%, ≥14% as high expression, and <14% as low expression.

### Statistical methods

SPSS 26.0 software and R software (version 4.1.2) were used for statistical analyses. The count data were analyzed using the chi-square test or Fisher’s exact probability method, and the measurement data were analyzed using the Kruskal–Wallis test. Univariate and multivariate logistic regression analyses were used to identify the independent predictors of the molecular subtype of breast cancer. The results were expressed as odds ratios (ORs) and 95% confidence intervals (CIs), and the regression coefficients of the regression model were displayed using the nomogram. AUC was calculated to evaluate the diagnostic efficacy of the nomogram. *P* value of less than 0.05 was considered statistically significant.

## Results

### Clinical data of the patients

A total of 363 breast cancer patients were included in this study, and 254 breast cancer patients were finally included, including 148 cases of HR+/HER2− breast cancer, 57 cases of HER2+ breast cancer, and 49 cases of Triple-Negative Breast Cancer (TNBC). The clinical and pathological details of the three breast cancer subtypes are provided in **Supplementary Table 5**.

### Analysis of MRI semantic features

There were significant differences between the molecular subtypes of breast cancer and tumor location, length, margin, enhancement characteristics, and peritumoral edema (*P <*0.05) (**Supplementary Table 3**).

As shown in **Table 1**, tumor length, edge enhancement, and peritumoral edema were independent risk factors for TNBC (*P* = 0.004, 0.005, and 0.032, respectively). Tumor length, edge spiculation, and peritumoral edema were independent risk factors for HR+/HER2− breast cancer (*P* = 0.002, 0.005, and 0.001, respectively). Peritumoral edema was an independent risk factor for HER2+ breast cancer (*P* = 0.039).

**Table 1:** Analysis of semantic features of clinical radiology in different molecular subtypes of breast cancer.

	Univariate analysis		Multivariate analysis	
	OR (95%CI)	*P-*value	OR (95%CI)	*P*-value
TNBC *vs.* no TNBC				
Location		0.016*		0.114
Upper Uter	1		1	
Lower Outer	0.630 (0.233–1.704)	0.363	0.723 (0.245–2.137)	0.723
Upper Inner	0.213 (0.077–0.589)	0.003	0.285 (0.098–0.829)	0.021
Lower Inner	0.341 (0.095–1.230)	0.100	0.316 (0.079–1.259)	0.102
Other Regions	1.229 (0.497–3.041)	0.656	1.020 (0.360–2.887)	0.970
Tumor length		<0.001*		0.004*
≤2 cm	1		1	
>2 cm	5.832 (2.900–11.728)		3.285 (1.465–7.367)	
Burr on edge		0.056		
No	1			
Yes	0.491 (0.237–1.018)			
Edge enhancement		<0.001*		0.005*
No	1		1	
Yes	4.746 (2.217–10.161)		3.504 (1.459–8.418)	
Peritumoral edema		<0.001*		0.032*
No	1		1	
Yes	5.027 (2.603–9.709)		2.357 (1.078–5.157)	
HR+/HER2− *vs.* others				
Location		0.042*		0.059
Upper Outer	1		1	
Lower Outer	1.425 (0.638–3.186)	0.388	1.450 (0.579–3.627)	0.428
Upper Inner	2.716 (1.411–5.230)	0.003	2.606 (1.243–5.460)	0.011
Lower Inner	2.217 (0.907–5.421)	0.081	2.587 (0.955–7.005)	0.062
Other Regions	1.571 (0.678–3.637)	0.292	2.637 (0.978–7.108)	0.055
Tumor length		<0.001*		0.002
≤2 cm	1		1	
>2 cm	0.227 (0.133–0.388)		0.380 (0.203–0.711)	
Burr on edge		0.001*		0.005*
No	1		1	
Yes	2.532 (1.447–4.431)		2.470 (1.313–4.646)	
Edge enhancement		0.008*		0.144
No	1		1	
Yes	0.368 (0.176–0.769)		0.524 (0.221–1.246)	
Peritumoral edema		<0.001*		0.001*
No	1		1	
Yes	0.179 (0.101–0.320)		0.305 (0.156–0.569)	
Her-2+ *vs.* others				
Location		0.558		
Upper Outer	1			
Lower Outer	0.960 (0.382–2.410)	0.931		
Upper Inner	0.800 (0.385–1.662)	0.550		
Lower Inner	0.823 (0.298–2.271)	0.707		
Other Regions	0.332 (0.093–1.194)	0.091		
Tumor length		0.119		
≤2 cm	1			
>2 cm	1.605 (0.886–2.907)			
Burr on edge		0.041*		0.077
No	1		1	
Yes	0.491 (0.248–0.972)		0.536 (0.268–1.071)	
Edge enhancement		0.421		
No	1			
Yes	0.682 (0.268–1.733)			
Peritumoral edema		0.019*		0.039*
No	1		1	
Yes	2.069 (1.124–3.806)		1.915 (1.033–3.551)	

*P indicates that the difference is statistically significant.

The diagnostic efficacy of MRI semantic features in identifying molecular subtypes of breast cancer is as follows: The AUC of TNBC was 0.78 (95%CI: 0.70–0.85). The AUC of HR+/HER2− was 0.74 (95%CI: 0.66–0.82). The AUC of HER2+ was 0.58 (95%CI: 0.50–0.67).

### Radiomics model

#### Radiomics model of tumor based on different sequences

A logistic regression classifier was used to establish a prediction model. A total of 1,130 features were extracted from the T2WI, DWI, and DCE sequences. After dimensionality reduction, 10 target features were retained for each sequence. In the task of identifying TNBC, HR+/HER2−, and HER2+ breast cancer, the top-ranked features in radiomics feature coefficients were Wavelet-LLH_glszm_ LargeAreaHighGrayLevelEmphasis, log-sigma-4-0-mm-3D_glcm_JointEnergy, and log-sigma-4-0-mm-3D_gldm_Large dependenceHighGrayLevelEmphasis.

**Table 2** shows that in the task of predicting molecular subtypes of breast cancer, the logistic regression model based on DCE radiomics features had the best prediction performance, with AUC values of 0.80, 0.78, and 0.76 in the training set and AUC values of 0.78, 0.79, and 0.72 in the validation set. For TNBC and HR+/HER2− breast cancer, the radiomics feature prediction model based on DWI outperformed the radiomics feature prediction model based on T2WI. Conversely, for HER2+ breast cancer, the radiomics feature prediction model based on T2WI demonstrated superior performance compared with the model based on DWI.

**Table 2:** Diagnostic performance of different MR sequence radiomics models in differentiating molecular subtypes of breast cancer.

	Training set				Validation set			
	AUC (95%CI)	Sens.	Spec.	Acc.	AUC (95%CI)	Sens.	Spec.	Acc.
TNBC *vs.* no TNBC								
T_2_WI	0.73 (0.64–0.82)	0.64	0.74	0.67	0.68 (0.53–0.84)	0.84	0.53	0.78
DWI	0.73 (0.64–0.82)	0.66	0.74	0.67	0.72 (0.58–0.86)	0.68	0.73	0.69
DCE	0.80 (0.72–0.88)	0.64	0.82	0.67	0.78 (0.62–0.93)	0.69	0.80	0.71
HR+/HER2− *vs.* others								
T_2_WI	0.75 (0.67–0.82)	0.82	0.55	0.67	0.67 (0.54–0.8)	0.59	0.80	0.71
DWI	0.70 (0.63–0.78)	0.74	0.61	0.67	0.75 (0.64–0.86)	0.75	0.73	0.74
DCE	0.78 (0.71–0.85)	0.66	0.78	0.73	0.79 (0.69–0.9)	0.75	0.80	0.78
Her-2+ *vs.* others								
T_2_WI	0.75 (0.67–0.82)	0.60	0.90	0.67	0.64 (0.47–0.80)	0.93	0.41	0.82
DWI	0.75 (0.66–0.83)	0.60	0.83	0.65	0.55 (0.39–0.72)	0.62	0.59	0.61
DCE	0.76 (0.68–0.84)	0.75	0.70	0.74	0.72 (0.57–0.86)	0.62	0.82	0.66

Sens. stands for sensitivity. Spec. stands for specificity. Acc. stands for accuracy.

#### Radiomics model of different peritumoral ranges based on DCE sequence

Under the DCE sequence, 1,130 features were extracted from each peritumoral region, combined with intratumoral radiomics features, and 10 target features were retained after dimensionality reduction. In the task of identifying TNBC, HR+/HER2−, and HER2+ breast cancer, the top-ranked features in the radiomics feature coefficients were log-sigma-4-0-mm-3D_ngtdm_Contrast, log-sigma-6-0-mm-3D_glcm_ldn, and wavelet-LHL_glcm_Correlation.

**Table 3**; **Figure 4** show the prediction performance of peritumoral features obtained with different peritumoral region sizes (3 mm, 6 mm, 9 mm, and 12 mm) in the training and validation sets. Among the three groups of breast cancer molecular subtypes, the AUC of the 6 mm peritumor model was the highest (training set: 0.82, 0.79, and 0.76; validation set: 0.80, 0.80, and 0.78, respectively). Moreover, after combining tumor and peritumoral features, it was found that the AUC of the combination model with peritumoral 6mm was the highest (training set: 0.92, 0.86, and 0.84; validation set: 0.85, 0.84, and 0.82). Among the radiomics models, it had the best performance in identifying TNBC (training set: 0.92, validation set: 0.85).

**Table 3:** Diagnostic efficacy of DCE-MRI peritumoral and tumor + peritumoral combined radiomics models in differentiating molecular subtypes of breast cancer.

	Training set				Validation set			
	AUC (95%CI)	Sens.	Spec.	Acc.	AUC (95%CI)	Sens.	Spec.	Acc.
TNBC *vs.* no TNBC								
Peritumoral								
Peri-3 mm	0.80 (0.71,0.89)	0.64	0.82	0.68	0.80 (0.67,0.93)	0.76	0.80	0.77
Peri-6 mm	0.82 (0.74,0.90)	0.67	0.85	0.71	0.80 (0.67,0.92)	0.69	0.80	0.71
Peri-9 mm	0.79 (0.70,0.87)	0.76	0.71	0.75	0.76 (0.60,0.91)	0.85	0.67	0.82
Peri-12 mm	0.76 (0.65,0.86)	0.84	0.62	0.80	0.75 (0.61,0.89)	0.60	0.73	0.62
Tumor + Peritumoral								
Com-3 mm	0.90 (0.84,0.95)	0.81	0.85	0.82	0.81 (0.68,0.93)	0.84	0.60	0.79
Com-6 mm	0.92 (0.86,0.98)	0.94	0.79	0.92	0.85 (0.76,0.94)	0.77	0.73	0.77
Com-9 mm	0.83 (0.74,0.91)	0.90	0.65	0.85	0.80 (0.66,0.94)	0.92	0.67	0.87
Com-12 mm	0.82 (0.75,0.90)	0.80	0.74	0.79	0.77 (0.63,0.92)	0.82	0.60	0.78
HR+/HER2− *vs.* others								
Peritumoral								
Peri-3 mm	0.81 (0.75,0.87)	0.82	0.65	0.72	0.78 (0.67,0.89)	0.88	0.60	0.71
Peri-6 mm	0.79 (0.72,0.85)	0.64	0.80	0.73	0.80 (0.70,0.89)	0.81	0.71	0.75
Peri-9 mm	0.78 (0.71,0.85)	0.78	0.66	0.71	0.77 (0.66,0.87)	0.72	0.71	0.71
Peri-12 mm	0.77 (0.70,0.84)	0.61	0.78	0.71	0.74 (0.63,0.85)	0.84	0.58	0.69
Tumor + Peritumoral								
Com-3 mm	0.80 (0.74,0.87)	0.80	0.73	0.76	0.83 (0.74,0.93)	0.66	0.91	0.81
Com-6 mm	0.86 (0.80,0.91)	0.78	0.83	0.81	0.84 (0.74,0.93)	0.72	0.89	0.82
Com-9 mm	0.80 (0.73,0.86)	0.78	0.71	0.74	0.80 (0.69,0.90)	0.78	0.82	0.81
Com-12 mm	0.78 (0.72,0.85)	0.61	0.83	0.73	0.76 (0.65,0.87)	0.78	0.71	0.74
Her-2+ *vs.* others								
Peritumoral								
Peri-3 mm	0.77 (0.7,0.84)	0.62	0.90	0.68	0.73 (0.59,0.86)	0.83	0.59	0.78
Peri-6 mm	0.76 (0.67,0.84)	0.62	0.80	0.66	0.78 (0.66,0.90)	0.63	0.88	0.69
Peri-9 mm	0.73 (0.64,0.82)	0.58	0.83	0.63	0.70 (0.56,0.83)	0.65	0.76	0.68
Peri-12 mm	0.73 (0.64,0.81)	0.54	0.85	0.61	0.64 (0.50,0.77)	0.52	0.82	0.58
Tumor + Peritumoral								
Com-3 mm	0.81 (0.72,0.88)	0.87	0.63	0.81	0.75 (0.62,0.86)	0.70	0.71	0.70
Com-6 mm	0.84 (0.73,0.90)	0.83	0.67	0.80	0.82 (0.70,0.93)	0.58	0.87	0.60
Com-9 mm	0.77 (0.70,0.85)	0.62	0.90	0.68	0.73 (0.59,0.86)	0.83	0.59	0.78
Com-12 mm	0.76 (0.67,0.84)	0.54	0.85	0.61	0.70 (0.56,0.83)	0.85	0.47	0.77

“Peri-” represents peritumoral features. “Com-” represents the combination of tumor and peritumoral features. Sens. stands for sensitivity. Spec. stands for specificity. Acc. stands for accuracy.

**Figure 4:**
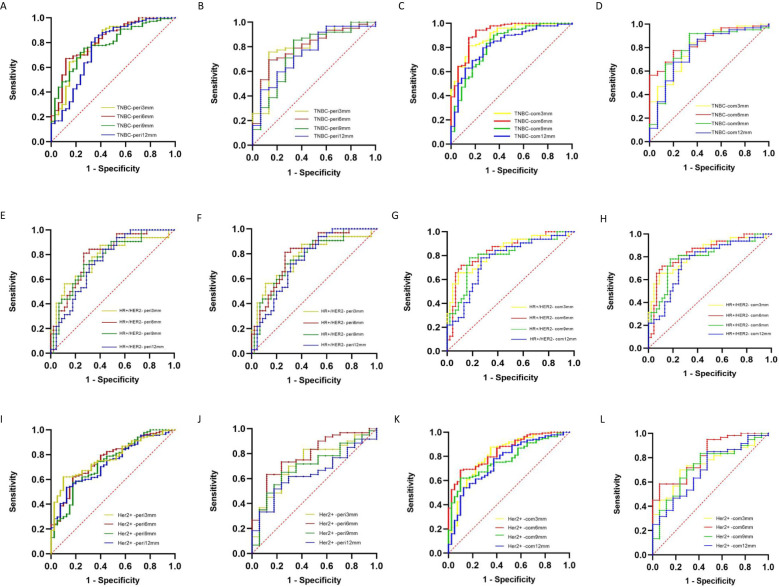
ROC curves of DCE-MRI peritumoral and tumor + peritumoral radiomics models for differentiating molecular subtypes of breast cancer **(A, B)** TNBC peritumoral model training and validation sets; **(C, D)** TNBC tumor + peritumoral model training and validation sets; **(E, F)** HR+/HER2− peritumoral model training and validation sets; **(G, H)** HR+/HER2− tumor + peritumoral model training and validation sets; **(I, J)** HER2+ peritumoral model training and validation sets; **(K, L)** HER2+ tumor + peritumoral model training and validation sets.

### Nomogram

#### Radiomics score calculation results

The Com-6 mm radiomics model had the best performance in predicting the molecular subtypes of breast cancer, and the radiomics score was calculated based on the combined model.

**Figure 5:**
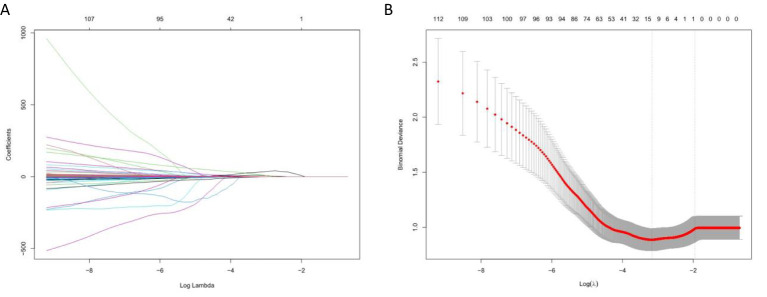
Selection of radiomics features using LASSO algorithm. **(A)** LASSO coefficient curve of radiomics features, each colored line represents the coefficient of each feature, and the unimportant feature coefficients were compressed to zero by adjusting λ; **(B)** Tenfold cross-validation was used to select the best performance radiomics feature map: the lower abscissa is the Log (λ) value, and the upper abscissa is the number of features after LASSO dimension reduction corresponding to the Log (λ) value.

A total of 2,260 highly reproducible features were analyzed by univariate regression analysis and the LASSO algorithm, and ten-fold cross-validation was used. Finally, 14, 14, and 13 optimal features with nonzero coefficients were selected for TNBC, HR+/HER2−, and HER2+ breast cancer, respectively (**Table 4** and **Figure 5**).

**Table 4:** Radiomics features and coefficients after LASSO dimensionality reduction.

Radiomics feature	Coefficient
TNBC	
original_shape_Sphericity (A1)	−0.299255
log-sigma-4-0-mm-3D_glcm_Idn (A2)	9.4466084
log-sigma-6-0-mm-3D_gldm_DependenceVariance (A3)	0.0088827
log-sigma-6-0-mm-3D_glcm_Idn(A4)	32.181428
log-sigma-6-0-mm-3D_firstorder_Skewness (A5)	0.3764279
plus original_shape_Maximum2DDiameterRow (A6)	0.000446
plus log-sigma-2-0-mm-3D_glcm_Correlation (A7)	0.7899561
plus log-sigma-2-0-mm-3D_firstorder_Kurtosis (A8)	−0.057933
plus log-sigma-2-0-mm-3D_glszm_SmallAreaLowGrayLevelEmphasis (A9)	−0.390269
plus wavelet-HLL_firstorder_Skewness (A10)	−0.65764
plus wavelet-LHL_glcm_Idn (A11)	13.439343
plus wavelet-HLH_glcm_ClusterShade (A12)	0.0024224
plus wavelet-HHL_glszm_GrayLevelNonUniformity (A13)	0.0005826
plus original_glszm_LargeAreaHighGrayLevelEmphasis (A14)	−0.00000269
HR+/HER2−	
log-sigma-2-0-mm-3D_glcm_Idn (B1)	−7.315145
llog-sigma-2-0-mm-3D_glszm_SmallAreaLowGrayLevelEmphasisn (B2)	−11.58611
log-sigma-4-0-mm-3D_firstorder_Skewness (B3)	−0.431533
log-sigma-6-0-mm-3D_glcm_Idn (B4)	−33.971521
wavelet-LHL_glcm_Correlation (B5)	0.514326
wavelet-LHL_firstorder_Skewness (B6)	0.078149
wavelet-LLL_glcm_Idmn (B7)	−1.034987
wavelet-LLL_firstorder_Kurtosis (B8)	−0.093055
original_glcm_Imc2 (B9)	0.456248
plus original_shape_Elongation (B10)	1.435092
plus wavelet-LLH_glcm_Idmn (B11)	−0.027667
plus wavelet-LLH_glcm_Idn (B12)	−0.193637
plus wavelet-LLL_gldm_LargeDependenceLowGrayLevelEmphasis (B13)	0.229736
plus wavelet-LLL_firstorder_Kurtosis (B14)	0.020340
HER2+	
original_shape_Elongation	−0.207434
log-sigma-2-0-mm-3D_glcm_Idn	0.0319403
log-sigma-2-0-mm-3D_firstorder_Kurtosis	0.0130694
log-sigma-2-0-mm-3D_glszm_SmallAreaLowGrayLevelEmphasis	0.4562349
wavelet-LHL_glcm_Correlation	−0.4332202
wavelet-LLL_firstorder_Kurtosis	0.02348109
original_glcm_Imc2	−0.6628339
plus log-sigma-2-0-mm-3D_glcm_ClusterShade	−0.000004
plus log-sigma-4-0-mm-3D_firstorder_Kurtosis	−0.01680067
plus wavelet-LHL_firstorder_Skewness	−0.01701304
plus wavelet-LHH_firstorder_Skewness	0.03400333
plus wavelet-HHH_firstorder_Kurtosis	0.00295915
plus wavelet-LLL_glrlm_LongRunLowGrayLevelEmphasis	−0.4477343

#### Construct the nomogram

1. To identify TNBC breast cancer

**Figure 6:**
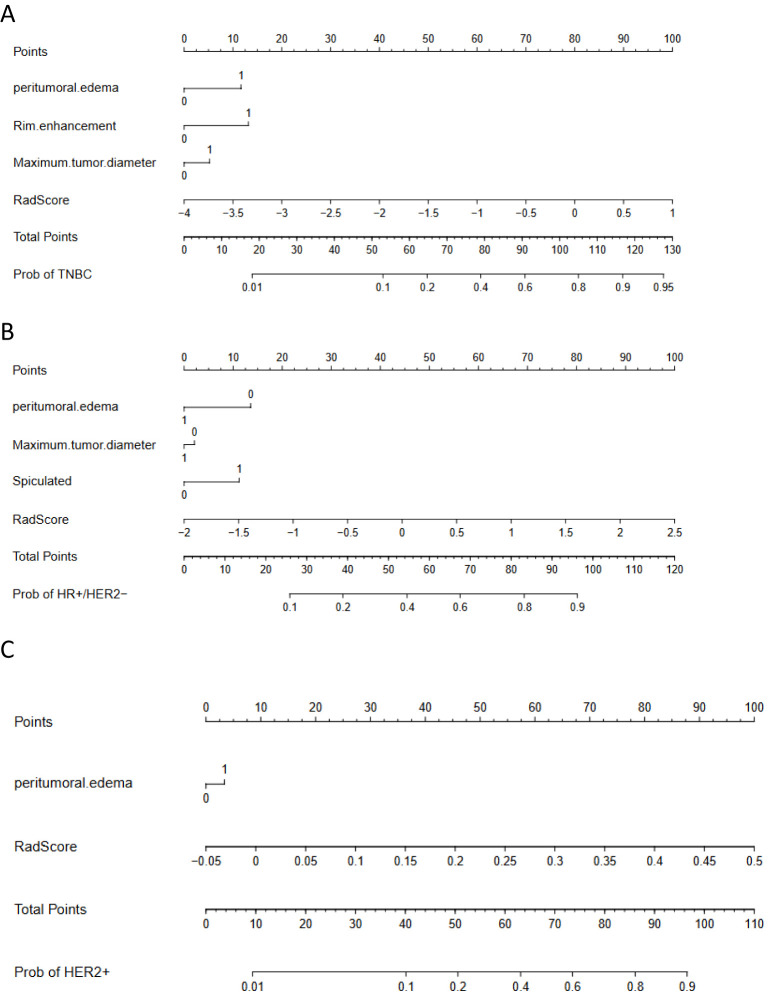
Nomograms. **(A)** to identify TNBC breast cancer. The nomogram was composed of peritumoral edema, rim enhancement, tumor length and radiomics score. Peritumoral edema: 0 = absent, 1 = present; Ring enhancement: 0 = no, 1 = yes; Tumor length: 0 = ≤2 cm, 1 = >2 cm. **(B)** to identify HR+/HER2− breast cancer. The nomogram was composed of peritumoral edema, tumor length, spiculation sign, and radiomics score. Peritumoral edema: 0 = absent, 1 = present; Tumor length: 0 = 2 cm, 1 = >2 cm; Spiculation: 0 = none, 1 = yes. **(C)** to identify HER2 + breast cancer. The nomogram consisted of peritumoral edema and radiomics score. Peritumoral edema: 0 = none, 1 = presence.


Independent predictors (tumor length, edge enhancement, and peritumoral edema) combined with the Rad-score were used to construct a nomogram. The AUC of the constructed nomogram in the training and validation sets were 0.848 (95%CI: 0.778–0.918) and 0.910 (95%CI: 0.840–0.979), respectively (**Figure 6**). The Rad-score was calculated using Equation 1.

(1) $$\begin{array}{c} Radscore=-53.55967+(-0.299255)\times A1+9.4466084\times A2+0.008827\times A3\\ +32.181428\times A4+0.3764279\times A5+0.00446\times A6+0.7899561\times A7\\ +(-0.057933)\times A8+(-0.390269)\times A9+(-0.65764)\times A10\\ +13.439343\times A11+0.0024224\times A12+0.0005826\times A13\\ +(-0.000000269)\times A14 \end{array}$$

2. To identify HR+/HER2− breast cancer

Independent predictors (tumor length, edge spiculation, and peritumoral edema) combined with the Rad-score were used to construct a nomogram. The AUC of the constructed nomogram in the training and validation sets were 0.834 (95%CI: 0.776–0.892) and 0.909 (95%CI: 0.839–0.978), respectively (**Figure 6**). The Rad-score was calculated using Equation 2.


(2) $$\begin{array}{c} Radscore=38.7805996+(-7.315145)\times B1+(-11.58611)\times B2\\ +(-0.431533)\times B3+(-33.671521)\times B4+0.514326\times B5\\ +0.078149\times B6+(-1.034987)\times B7+(-0.093055)\times B8\\ +0.456248\times B9+1.435092\times B10+(-0.027667)\times B11\\ +(-0.193637)\times B12+0.229736\times B13+0.020340\times B14 \end{array}$$

3. To identify HER2+ breast cancer

An independent predictor (peritumoral edema) combined with the Rad-score was used to construct a nomogram. The AUC of the constructed nomogram in the training and validation sets were 0.802 (95%CI: 0.730–0.873) and 0.845 (95%CI: 0.742–0.948), respectively (**Figure 6**). The Rad-score was calculated using Equation 3.


(3) $$\begin{array}{c} Radscore=0.8623712+(-0.207434)\times C1+0.0319403\times C2+0.0130694\\ \times C3+0.4562349\times C4+(-0.4332202)\times C5+0.02348109\\ \times C6+(-0.6628339)\times C7+(-0.000004)\times C8\\ +(-0.01680067)\times C9+(-0.01701304)\times C11+0.00295915\\ \times C12+(-0.4477343)\times C13 \end{array}$$

## Discussion

This study aimed to investigate the performance of radiomics and nomogram models based on multiple MRI sequences for the noninvasive prediction of molecular subtypes of breast cancer.

We analyzed the semantic features of breast cancer on MRI and found that edge spiculation, edge enhancement, and peritumoral edema correlated with the molecular subtype of breast cancer. Previous studies have also found that tumor edge enhancement is closely related to the overexpression of vascular endothelial growth factor and tumor hypoxia, which is common in fast-growing tumors and is related to tumor size, grade, ER and/or PR expression, Ki-67 expression, lymph node status, and DNA S-phase percentage (12, 13). TNBC is the most aggressive breast cancer. We also conclude that edge enhancement is a predictor. Tumor edge spiculation is associated with positive ER and PR expression, negative HER2 and Epidermal Growth Factor Receptor (EGFR) expression, and lymph node metastasis (14), and we believe that it is a predictor of HR+/HER2− breast cancer. Peritumoral edema is mainly caused by increased endothelial permeability of tumor neovascularization and peritumoral cytokine release, and is commonly seen in triple-negative breast cancer and HR-deficient breast cancer (15–17). We support the conclusion that peritumoral edema is a positive predictor for TNBC and HER2+ subtype breast cancer, and a negative predictor for HR+/HER2− subtype breast cancer. The results of this study not only support the conclusions of previous studies but also transform the traditional qualitative analysis method of semantic features into a quantitative analysis method. By constructing the nomogram model, the weight proportion of the semantic features in the construction of the prediction model was accurately quantified.

Studies have reported that DCE sequences reflect more detailed biological information of tumors by analyzing the hemodynamic characteristics of tumors (17, 18), and that texture features are correlated with the levels of multiple biomarkers, such as estrogen receptor (ER), progesterone receptor (PR), and HER2 (19, 20). Our study compared radiomics models based on T2WI, DWI, and DCE sequences, and the results showed that the DCE-based model had the highest diagnostic efficiency (AUC = 0.910), especially in the differentiation of TNBC and non-TNBC breast cancer, which may be related to the higher heterogeneity of TNBC breast cancer. The heterogeneity of tumor morphology and contrast enhancement in the DCE sequence can better reflect pathophysiological characteristics, such as tumor proliferation and angiogenesis (21). The AUC result of this validation set was better than that of previous models established to differentiate TNBC from non-TNBC breast cancer, such as the study by Zhang et al. (22) (AUC =0.879) and Zhang et al. (23) (AUC = 0.890), which reflects that we have a superior model scheme.

In addition to the tumor body, the radiomics features of the surrounding areas of breast cancer are also of great significance. Based on DWI images, Fan et al. (24) explored the relationship between radiomics features of tumors and their surrounding areas and molecular subtypes of breast cancer, and found that the model established when the peritumoral area was 5 mm had the best prediction performance. Zhang et al. (22) used peritumoral radiomics features based on DCE-MRI to establish a molecular classification model for breast ductal carcinoma *in situ*. The best peritumoral area was 6 mm for differentiating between TNBC and non-TNBC, HR+/HER2−, and non-HR+/HER2−. The optimal peritumoral area for distinguishing HER2+ cells from non-HER2+ cells was 8 mm. Hao et al. (8) studied the distance of 4 mm around the tumor and established a preoperative molecular classification model of breast cancer. Based on DCE-MRI images, we compared various peritumoral ranges (3 mm, 6 mm, 9 mm, and 12 mm) and established multiple radiomics logistic regression models. We found that the peritumoral 6 mm radiomics feature model had the best performance in identifying molecular subtypes of breast cancer, and the performance was better than that of previous studies. In addition, our study included a variety of histological types of breast cancer, and the conclusions were more generalized and applicable.

In this study, the proportion of high-order features extracted from T2WI, DWI, and DCE sequences for the differentiation of the three groups of breast cancer molecular subtypes was much higher than that of the low-order features. Higher-order features reflect the consistency between texture roughness and tumor texture images, which is beneficial for better predicting the heterogeneity within the tumor and the subtle differences in gray texture features, and provides more information for the evaluation of breast cancer molecular typing diagnosis. As an important part of high-order features, wavelet transform analyzes the local time and spatial frequency, extracts high-frequency and low-frequency signals in the image extensively and effectively, and reflects the texture changes of the image more carefully and comprehensively. The Gabor transform performed by the Gabor filter based on the wavelet transform can be used to solve the lack of localization-analysis ability of the Fourier transform and the analysis ability of non-stationary signals.

Previous studies have also found that wavelet features contain more detailed information about breast cancer and are a key component in radiomics model construction (25). Braman et al. (26) found that Gabor features are of great significance in the molecular classification of breast cancer, which can improve the ability to distinguish HER2+ from other breast cancers (such as TNBC). In this study, wavelet features demonstrated good predictive power for HER2+ breast cancer and can be used to quantify tumor heterogeneity comprehensively and broadly at different spatial scales and directions. In this study, the wavelet features demonstrated good predictive power for HER2+ breast cancer and can be used to quantify tumor heterogeneity comprehensively and broadly at different spatial scales and directions. The mixture of a variety of low- and high-order features has heterogeneous information complementary values for distinguishing the molecular subtypes of breast cancer. Niu et al. (27) found that shape features could distinguish TNBC from other molecular types of breast cancer. In this study, shape feature-sphericity was also of great significance in identifying TNBC. It is the only low-order feature retained after the LASSO dimension reduction, and its absolute value of the feature coefficient is at the 8th place.

In recent years, an increasing number of studies have developed nomograms to intuitively assist the clinical decision-making process and make the treatment strategy for breast cancer patients more convenient, accurate, and personalized. Kim et al. (28) developed a nomogram based on MRI and clinical-pathological variables to predict breast cancer Polymerase Chain Reaction (PCR), which showed higher efficiency than a single clinical-pathological model. Yu et al. (29) developed a nomogram combining radiomics and clinical features for preoperative prediction of axillary lymph node metastasis and disease recurrence risk in early breast cancer. Decision curve analysis showed that the clinical-radiomics nomogram had better predictive performance than clinical or radiomics alone. In this study, a nomogram based on semantic features and tumor + peritumoral 6 mm radiomics score was constructed to identify molecular subtypes of breast cancer, and the diagnostic efficiency was significantly higher than that of MRI semantic feature analysis and radiomics model alone. In this study, the Rad-score had the widest predictive score and the largest contribution in the nomogram and was the most important independent factor for the identification of molecular subtypes of breast cancer. Clinicians can perform nomogram analyses based on individual differences and available information, which provides methodological information for the prediction of molecular subtypes of breast cancer.

This study has several limitations. First, it should be noted that the retrospective nature of the study introduces a certain degree of selection bias. Second, it is important to acknowledge that this study was conducted at a single center, which may limit the generalizability of the findings. Therefore, future multicenter studies are required to validate the radiomics model proposed in this study. Lastly, while peritumoral radiomics models were successfully established using DCE sequences, similar models utilizing T2WI and DWI sequences have not been developed. Although the DCE sequence outperforms T2WI and DWI sequences in predicting breast cancer molecular subtypes without compromising the final results, it is also essential to consider experimental integrity. In future research endeavors, we aim to delve deeper into extracting valuable information from the T2WI and DWI sequences.

## Conclusions

The nomogram based on independent predictors combined with intratumoral and peritumoral radiomics scores can be used as an auxiliary diagnostic tool for the molecular subtype prediction of breast cancer.

## Data Availability

The original contributions presented in the study are included in the article/**Supplementary Material**. Further inquiries can be directed to the corresponding authors.
