# Supplementary material for: The value of intratumoral and peritumoral radiomics features based on multiparametric MRI for predicting molecular staging of breast cancer
Source: Front Oncol. 2025 Mar 11;15:1379048. doi: 10.3389/fonc.2025.1379048 (PMC11933106; doi:10.3389/fonc.2025.1379048)
Supplement: Supplementary file 1 [file Table1.docx]

Table 1 Inclusion and exclusion criteria of cases

| Criteria |  |
| --- | --- |
| Inclusion | (1) complete imaging data and clinical history data;  (2) Before MRI examination, the patient did not receive any intervention measures such as puncture, surgery, radiotherapy, chemotherapy, etc.  (3) The interval between MRI examination and surgery was ≤2 weeks, and histopathological and immunohistochemical staining was obtained. |
| Exclusion | (1) poor image quality (n=44);  (2) non-mass-like breast cancer (n=37);  (3) multifocal lesions in the ipsilateral breast (n=28). |

Table 2 MR Scan sequence and related parameters

| Sequence | TR(ms) | TE(ms) | Thick (mm) | FOV(mm) | Contrast agent | Contrast agent dosage |
| --- | --- | --- | --- | --- | --- | --- |
| T_1_WI | 6-600 | Min Full-3 | 4-5 | 300-360×300-360 |  |  |
| FS-T_2_WI | 4000-4500 | 60-90 | 4-5 | 300-360×300-360 |  |  |
| DWI | 4100-12800 | Minimum-80 | 4-5 | 300-380×300-380 |  |  |
| DCE-MRI | 4-8 | Minimum-12 | 3 | 320-360×320-360 | GD-dtpa | 0.2mmol/kg |

Table 3 Analysis of MRI semantic features

| Semantic features | Notes |
| --- | --- |
| Tumor location | Within a quadrant, quadrant goes up outside, on quadrant, inside the quadrant, other area (3, 6, 9 and 12 o 'clock direction and the central gland area) |
| Tumor length (cm) | the maximum diameter of the tumor on the horizontal axis |
| The fiber glands (fibroglandular tissue, FGT) constitute a classification | Is divided into four type: on T1WI sequence, type a: almost all of fat (< 25% of the mammary gland containing gland tissue); B: in the distribution of the fiber glands (25% - 50% of the mammary gland containing gland tissue); Type c: heterogeneous distribution of fibrous glandular tissue (51%-75% of the breast contains glandular tissue); Type d: dense fibrous glandular tissue (> 75% of the breast contains glandular tissue) |
| Background parenchymal enhancement (BPE) | It refers to the normal enhancement of fibrous glandular tissue, which appears about 90s after enhancement, including slight enhancement (< 25% of glandular tissue enhancement), mild enhancement (25%-50% enhancement), moderate enhancement (50%-75% enhancement), and significant enhancement (> 75% enhancement) |
| Shape of tumor | Oval (including lobulated), round, irregular |
| Margin of the mass | Smooth, irregular and spiculated |
| Mass-like enhancement features | Homogeneous, heterogeneous and marginal enhancement |
| Time ‑ signal intensity curve (time intensity curve, TIC) classification | Rising type (Ⅰtype), platform (Ⅱ type) and outflows (Ⅲ type) |
| Peritumoral edema | Fluid-like high signal on FS-T2WI, which was different from the obvious tumor boundary without mass effect, was recorded as with and without edema |
| T2WI signal level | FS-degree of tumor signal on T2WI sequences, compared with the adjacent normal breast tissue signal, can be divided into low, and high signal, etc |

Table 4 Radiomics features

| Features | Notes |
| --- | --- |
| Shape features | The geometric characteristics and shape features of VOI were described； |
| First-order features | Reflect the distribution of voxel values in VOI, but do not involve the spatial arrangement of voxels. They are based on histogram properties, such as the mean, middle, maximum, minimum, skewness, kurtosis, etc； |
| Texture features | The intensity of spatially arranged voxels, which is obtained by calculating the statistical correlation between adjacent voxels and reflects the heterogeneity of lesions, include: a) gray-level co-occurrence matrix (glcm): In a certain range of distance, it is the basis of analyzing the spatial correlation of images to reflect the information of the gray level of voxels in terms of direction, adjacent interval, and variation amplitude. b) gray-level dependence matrix (gldm): it refers to the calculation of the probability density function of gray level difference distribution of a given image; c) gray-level size zone matrix (glszm) and gray-level run-length matrix (glrlm): The calculation process of the two methods is very similar, which quantifies the number of connected voxels with the same gray level in a specific direction. d) neighborhood gray-tone difference matrix (ngtdm): refers to the sum of the differences between the mean values of the voxels with different gray levels and the surrounding voxels; |
| Higher-order statistical features | such as Laplace transform and wavelet transform. |

Table 5 Different molecular classification of breast cancer patients with clinical and pathological data

| **Clinical Information** | HR+/HER2-  （n=148） | Her-2+  (n=57) | | | TNBC  (n=49) | *P* | |
| --- | --- | --- | --- | --- | --- | --- | --- |
| **Age (years)** | 54.01±11.12 | 52.93±11.08 | | | 54.65±12.35 | 0.604 | |
| **Menopausal status** |  |  | | |  | 0.850 | |
| Not in | 59（39.9%） | 24（42.1%） | | 18（36.7%） | | |  |
| Have gone through | 89（60.1%） | 33（57.9%） | | 31（63.3%） | | |  |
| **Family History** |  |  | | |  | 0.196 | |
| No | 139（93.8%） | | 53（93.0%） | 42（85.7%） | | |  |
| Have | 9（6.1%） | | 4（7.0%） | 7（14.3%） | | |  |
| **Tumor length** | 1.81±0.61 | 2.12±0.68 | | | 2.43±0.90 | 0.003* | |
| **Classification** |  |  | | |  | 0.003* | |
| Infiltrative type not otherwise specified | 143（97.3%） | | 50（87.7%） | 49（100.0%） | | |  |
| Mucinous carcinoma | 3（2.0%） | | 1（1.8%） | 0 | | |  |
| Invasive micropapillary carcinoma | 1（0.7%） | | 0 | 0 | | |  |
| Mixed carcinoma | 0 | | 1（1.8%） | 0 | | |  |
| High level nuclear ductal carcinoma in situ | 0 | | 5（8.8%） | 0 | | |  |
| **Axillary lymph node metastasis** |  |  | | |  | 0.005* | |
| No | 117（79.1%） | 33（57.9%） | | | 39（79.6%） |  | |
| Have | 31（20.9%） | 24（42.1%） | | | 10（20.4%） |  | |
| **Ki-67** |  |  | | |  | <0.001* | |
| ＜14 | 61（41.2%） | 4（7.0%） | | | 1（2.0%） |  | |
| ≥14 | 87（58.8%） | 53（93.0%） | | | 48（98.0%） |  | |

Note: **P* indicates that the difference is statistically significant.
